# Chern insulator with a nearly flat band in the metal-organic-framework-based Kagome lattice

**DOI:** 10.1038/s41598-019-50163-7

**Published:** 2019-09-24

**Authors:** Santu Baidya, Seungjin Kang, Choong H. Kim, Jaejun Yu

**Affiliations:** 1 Center for Correlated Electron Systems, Institute for Basic Science, Seoul, 08826 Korea; 2 Department of Physics and Astronomy, Seoul National University, Seoul, 08826 Korea; 3 Center for Theoretical Physics, Department of Physics and Astronomy, Seoul National University, Seoul, 08826 Korea

**Keywords:** Quantum Hall, Topological matter

## Abstract

Based on first-principles density-functional theory (DFT) calculations, we report that the transition-metal bis-dithiolene, *M*_3_C_12_S_12_ (*M* = Mn and Fe), complexes can be a two-dimensional (2D) ferromagnetic insulator with nontrivial Chern number. Among various synthetic pathways leading to metal bis-dithiolenes, the simplest choice of ligand, Benzene-hexathiol, connecting metal cations to form a Kagome lattice is studied following the experimental report of time-reversal symmetric isostructural compound Ni_3_C_12_S_12_. We show sulfur and carbon-based ligands play the key role in making the complexes topologically nontrivial. An unusual topological quantum phase transition induced by the on-site Coulomb interaction brings a nearly flat band with a nonzero Chern number as the highest occupied band. With this analysis we explain the electronic structure of the class *M*_3_C_12_S_12_ and predict the existence of nearly flat band with nonzero Chern number and it can be a fractional Chern insulator candidate with carrier doping.

## Introduction

The quantum Hall effect (QHE) is observed in two-dimensional (2D) electron systems under the presence of strong external magnetic field as a result of the formation of well-defined Landau levels^1,2^. Considering that the dissipationless states at sample edges is a consequence of the topologically nontrivial ground state of the Landau quantization^3,4^, however, there have been numerous proposals to realize the QHE without applying any magnetic field. Haldane^5^ first pointed out that a topologically nontrivial electronic structure with broken time reversal symmetry (TRS) in a 2D insulator can induce such quantized anomalous Hall effect (QAHE), where the quantized Hall conductance is determined by the topological character of the band structure, i.e., Chern number. Later, the idea has been extended to magnetically doped topological insulator thin films^6–10^.

Following the theoretical proposals that the magnetic order induced by the doped transition metal elements can lead to a topological electronic structure with a finite Chern number in 2D thin films, various attempts have been made to realize magnetic topological insulators and the observations of QAHE were reported^8,11–13^. These magnetic topological insulators have attracted a lot of attention for their potential applications to the low energy consumption electronics and spintronics devices^14,15^. However, since the realization was limited to an extremely low temperature of about 30 mK due to the small bulk energy gap and ferromagnetic (FM) Curie temperature^13^, there is a high demand for high temperature QAHE materials for their future applications.

So far, most of theoretical proposals for novel magnetic topological insulators, namely, Chern insulators, are based on the honeycomb-lattice platform and utilize the half-metallic band structure with Dirac bands^16–20^. Also, the co-doping of exisiting topological insulators^21,22^, the bulk ferromagnetic BaFe_2_(PO_4_)_2_^23^, the honeycomb structure stanene^24^, and niobate^25^ were suggested as candidates for Chern insulators. Further, the (111)-oriented growth of perovskite oxide heterostructures was proposed^26–28^. Apart from inorganic materials, however, Wang and coworkers suggested that a metal-organic compound, i.e., triphenyl-transition-metal (Mn_2_C_18_H_12_), in a 2D hexagonal lattice shows the QAHE with a nonzero Chern number^29^. Further, the successful synthesis of 2D metal-organic frameworks (MOFs) has opened a possiblity of numerous novel properties of 2D organic transition-metal chalcogen family^30,31^. This kind of two-dimensional system such as the honeycomb stanene^32^, arsenene^33^, and Bi/Sb(111) films^34^ could also host quantum spin Hall insulator phase. Also, there were proposals for the organometallic lattices with Fe, Cr and Co to exhibit half-metallic from first-principles calculations^35^. Although the single layer *π*-conjugated nickel-bis-dithiolene Ni_3_C_12_S_12_ was identified to have nontrivial topological states^29,36^, its realization still requires a precise control of oxidation state at the gapped Dirac point^37^. However, question remains on the choice of ligands and metal cations to carry out precise tuning of Fermi level to observe exotic properties.

A flat band, dispersionless band in the whole Brillouin zone, has attracted lots of attention since it can be an ideal playground to study strong correlation physics. Especially, a nearly flat band with a nontrivial Chern number was recently proposed as a promising candidate for the realization of fractional Chern insulators^38–42^. Kagome lattice with nearest hopping naturally provide flat bands due to the self-localization under destructive interference^43,44^. Then one also could expect a nearly flat band with nontrivial Chern number in the ferromagnetically ordered Kagome-lattice-based MOF.

The choice of transition metal cations in MOFs gives a subtle balance between strong correlation and spin-orbit coupling into the system and giving rise to a range of functionalized compounds with non-trivial topological electronic structure. The role of Mn and Fe is two fold, the time-reversal-symmetry breaking by rather large spin and giving rise to localized electronic bands with large spin-splitting near Fermi level. We propose a realization of Chern insulator in the Kagome-lattice-based MOF with the simplest choice of benzene-hexathiole (BHT) ligands with Mn and Fe as 3*d* transition metal cations for Chern insulators. We investigate the topological character of the band structures of 2D MOF kagome lattice of *π*-conjugated metal bis-dithiolene *M*_3_C_12_S_12_ (*M* = Mn and Fe), which can take an advantage of several important ingredients: the lattice symmetry, broken time-reversal symmetry, and reliable spin-orbit coupling. The flat band feature arising from the frustrated kagome lattice become a central part of the band structure across the Fermi level. Using density functional theoretical analysis we have shown that the 2D Kagome lattice of metal-bis dithiolene can show intrinsic anomalous Hall effect. The *non-typical* phase transition places the topologically nontrivial flat band just below the Fermi level in both Mn and Fe bis-ditholene complex. Both the complex show Ising type ferromanetic ordering with near room temperature magnetic *T*_*c*_ which could be very useful in spintronics application.

## Results and Discussion

**Figure 1:**
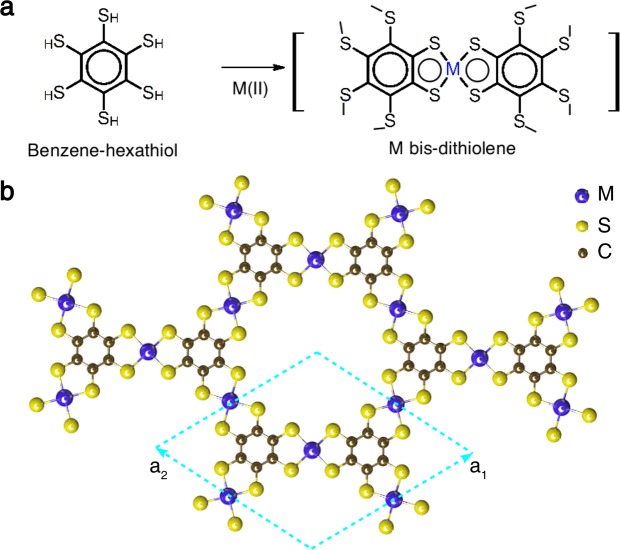
(**a**) The schematics of reaction to produce metal bis-dithiolene complex unit that makes network of metal cations (M) as Kagome lattice. (**b**) Crystal structure of single layer metal bis-dithiolene kagome lattice (MOF). a_1_ and a_2_ being inplane lattice constants.

Figure 1(a) shows a schematic illurstration of the metal (*M*) bis-dithiolene unit which supports a network of *M* cations in a 2D kagome lattice as shown in Fig. 1(b). The synthesis of the complex nanosheet consisting of *π*-conjugated nickel (Ni) bis-dithiolene has already been reported by Kambe *et al*.^30^. The network of *π*-conjugated metal bis-dithiolene in a chemical formula of *M*_3_C_12_S_12_ (*M* = Mn and Fe) forms a triangular lattice with the unit vectors ***a***_1_ and ***a***_2_, as drawn in Fig. 1(b). The *M* atom is coordinated by four S atoms. The ligand Benzene-hexathiol connects the *M* atoms forming two motifs, honeycomb motif and triangle motif, which gives rise to Kagome lattice for *M* atoms.

Figure 1(b) shows the schematic repeesenatation of the metal bis-dithiolene (M_3_C_12_S_12_). A full relaxation is carried out for the single-layer Manganese bis-dithiolene (MDT) Mn_3_C_12_S_12_ and iron bis-dithiolene (FDT) Fe_3_C_12_S_12_. In the primitive unit cell, there are three *M* atoms, twelve C atoms and twelve S atoms. The space group of the single-layer *M*_3_C_12_S_12_ is P6/mmm. The fully relaxed lattice constant is $a=14.971\,{\rm{\AA }}$ for Mn and $a=14.798\,{\rm{\AA }}$ for Fe, respectively. (The details of lattice constants and atom position coordinates are listed in the Supplementary Information). The relaxation is carried out the way to maintain *C*_3_ symmetry and inversion symmetry in the lattice. The plaquet of S around *M* atom is rectangular.

**Figure 2:**
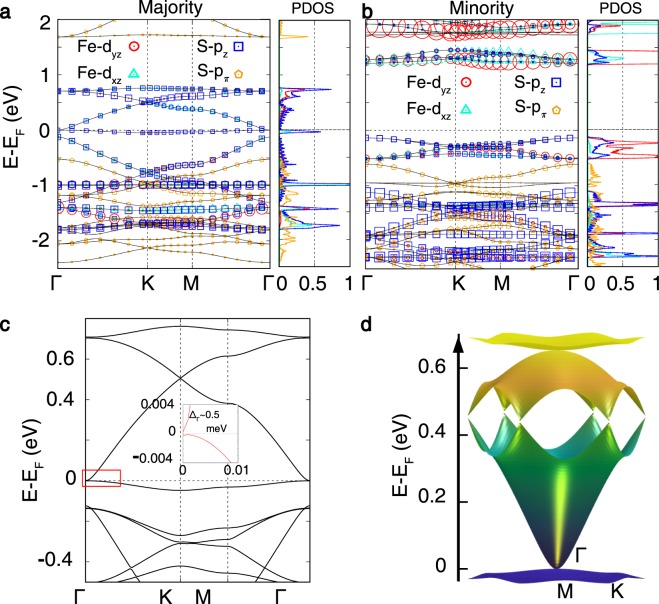
(**a**, **b**) PBE + *U* “fatband” and projected density of states of the Fe *d*_*xz*_, *d*_*yz*_ and S *p*_*z*_, *p*_*π*_ states within the energy manifold near Fermi level plotted for majority (**a**) and minority (**b**) spin channel. The size of symbols is proportional to character of states. The projected density of states (PDOS) in unit of eV/states/atom for the Fe *d*_*xz*_, *d*_*yz*_ and S *p*_*z*_, *p*_*π*_ states are plotted corresponding to both the spin channel representing relative positions of the states. (**c**) PBE + *U* + SOC band structure shows opening tiny band gap at $\Gamma$ point (shown in the inset). (**d**) Three dimensional PBE + *U* + SOC band structure of Mn_3_C_12_S_12_ (MDT) monolayer.

First we analyze the basic electronic and magnetic structures of *M*_3_C_12_S_12_ (*M* = Mn and Fe). Within the PBE + *U* method with *U* = 3 eV in vasp, the ground state of both systems is a half-metallic ferromagnet, Dirac cone at the Fermi level in the majority spin channel and insulating in the minority spin channel (see Fig. 2(a,b)). In the absence of spin-orbit coupling, the degeneracy at $\Gamma$ point is preserved. The inclusion of spin-orbit coupling using opens a tiny gap at the Fermi level about 0.5 meV lifting degeneracy at the $\Gamma$ point and the complex is an insulator (see Fig. 2(c)). A three-dimensional view of the electronic band structure of MDT is shown in Fig. 2(d)).

The main orbital characters near Fermi level are from Fe *d*_*xz*_, *d*_*yz*_ and S *p*_*z*_, *p*_*π*_ as projected within the energy manifold shown in the Fig. 2(a,b). The superexchange interaction between the two consequtive Fe atoms is mediated by the *π*-conjugated bonding due to molecular orbitals formed by Fe *d*_*xz*_, *d*_*yz*_ and S *p*_*z*_ orbitals (Fig. 2(a,b)). Whilst the band center of Fe *d*_*xz*_ and *d*_*yz*_ orbitals lies around −5 eV to −4 eV the tail part goes beyond Fermi level due to the large covalency with S *p* orbitals. The C *p*_*z*_ orbitals mainly dominate energy range just below Fermi level. The Fe *d*_*z*2_ is fully occupied around −3 eV in the majority spin channel due to non-bonding along *z* direction of the unitcell. The S *p*_*π*_ has a occupied bonding character lying around −1 eV in the majority spin channel while unoccupied antibonding character lies around 2 eV. On the other hand, the Fe *d* orbitals are unoccupied in the minority spin channel. Two holes at S *p*_*z*_ orbital in the minority spin channel contributes two electrons at the Fe site which makes the total spin at Fe to be $S=1$ (${d}^{6}{\underline{{\rm{L}}}}^{-2,\downarrow }$). Here, the notation “${d}^{6}{\underline{{\rm{L}}}}^{2\downarrow }$” represents a local electronic configuration of six *d*–electrons (*d*^6^) of Fe and two down-spin (↓) holes (${\underline{{\rm{L}}}}^{2}$) of S ligand. With the same analysis total spin at Mn site in MDT appears to be $S=\tfrac{3}{2}$ (${d}^{5}{\underline{{\rm{L}}}}^{-2,\downarrow }$).

The ferromagnetic ground state arises from the local moment of Mn and Fe. The formal oxidation state of Fe is 2+ with electronic configurations ${d}^{6}{\underline{{\rm{L}}}}^{-2,\downarrow }$. With the same analysis electronic configuration for Mn in MDT appears to be ${d}^{5}{\underline{{\rm{L}}}}^{-2,\downarrow }$. The *d* electron configuration of isolated Mn ion should be *d*^5^. Due to the molecular orbital formation with dithiolene ligands which contribute two “↓” spin electrons per connected Mn site the local electron spin at Mn site is ${d}^{5}{\underline{{\rm{L}}}}^{-2,\downarrow }$. The similar argument can be attributed for the electron configuration at Fe site ${d}^{6}{\underline{{\rm{L}}}}^{-2,\downarrow }$. It makes the molecular *t*_2*g*_ orbital at Mn site half-filled, $S=\tfrac{3}{2}$, which interacts with the nearest-neighbour Mn sites ferromagnetically through the antiferromagnetic interaction with dithiolene ligands to lift the spin-frustrated situation with out-of-plane orientation. This explanation would equally valid for FDT with spin at Fe *S* = 1. The calculated spin moments are 2.6 *μ*_*B*_ at the Fe site, −0.076 *μ*_*B*_ at the S site, −0.034 *μ*_*B*_ at the C site for FDT; 3.78 *μ*_*B*_ at the Mn site, −0.13 *μ*_*B*_ at the S site, −0.04 *μ*_*B*_ at C site for MDT.

The magnetic Curie temperature *T*_*c*_ is also calculated from DFT total energy comparison of various collinear magnetic configurations with considering nearest neighbor Heisenberg interaction. The effective Fe-Fe spin exchange interaction ${J}_{Fe-Fe}$ is computed to be 11.3 meV and Mn-Mn interaction ${J}_{Mn-Mn}$ is 4.2 meV. Using the values of exchange interactions and the total spin value of Fe $S=1$ and of Mn $S=3/2$ (both in classical spin limit), mean-field estimation of magnetic *T*_*c*_ is computed for FDT as ${T}_{c}^{{\rm{Fe}}}\sim 352\,{\rm{K}}$ and for MDT as ${T}_{c}^{{\rm{Mn}}}\sim 291\,{\rm{K}}$. We have also performed classical Monte-Carlo simulation to estimate *T*_*c*_ for both MDT and FDT by calculating specific heat and absolute magnetization as a function of temperature (see the Supporting Information). Our Monte Carlo simulation gives close but little less magnitude to the mean-field estimate of the *T*_*c*_ for MDT to be ${T}_{c}^{{\rm{Mn}}}\sim 234\,{\rm{K}}$ and for FDT to be ${T}_{c}^{{\rm{Fe}}}\sim 281\,{\rm{K}}$. The magnetic anisotroy energy is also calculated by comparing the energy of in-plane and out-of-plane collinear configurations. The out-of-plane configuration is 0.87 meV lower in energy compared to the in-plane order for FDT and the same for MDT is 1.9 meV.

**Figure 3:**
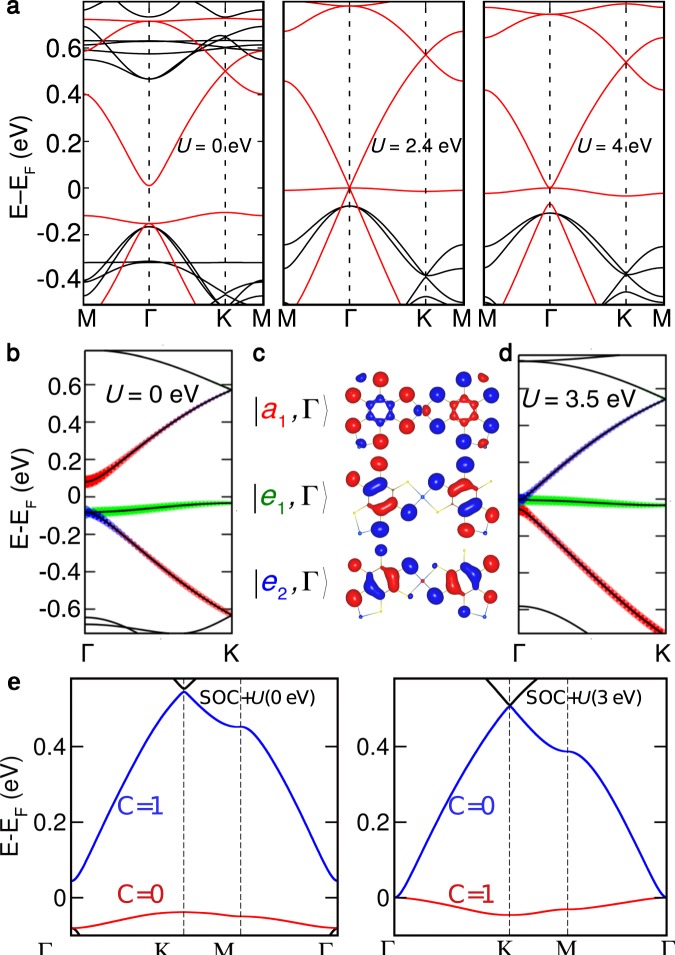
Strong correlation driven band inversion in single layer Fe_3_C_12_S_12_. (**a**) The PBE + *U* spin-polarized majority (red line) and minority (black) spin channel) band evolution with *U*. The majority spin channel bands shows similar to low-energy Kane model behaviour. (**b**) The PBE + *U* (*U* = 0 eV) band structure with weight of eigenstates at $\Gamma$ point, $|{a}_{1},\Gamma \rangle$ (red color), $|{e}_{1},\Gamma \rangle$ (green color) and $|{e}_{2},\Gamma \rangle$ (blue color) projected along $\Gamma -K$ direction in the BZ. (**c**) The shape of the molecular orbitals $|{a}_{1},\Gamma \rangle$, $|{e}_{1},\Gamma \rangle$ and $|{e}_{2},\Gamma \rangle$ at $\Gamma$ point. (**d**) The PBE + *U* (*U* = 3.5 eV) band structure with degenerate eigenstate exactly at the $\Gamma$ point and flat band at the Fermi level. The color of the wights of the MOs are now inverted. (**e**) Chern number of flat band in PBE + *U* + SOC calculations with *U* = 0 and 3 eV.

To highlight the uniqueness of electronic structure in the 2D MOF kagome lattice, we focus on the FDT complex in the rest of the paper, but the main features of the Mn_3_C_12_S_12_ (MDT) system are similar except the differences in band gap and bandwidths. In Fig. 3(a), we have shown the evolution of band structure of majority spin by increasing on-site *U*. It is interesting to note that at *U* = 3 eV there are *non-typical* four bands near the Fermi level (*E*_F_), out of which top three bands displaying a typical kagome band dispersion. Among the four bands, the top and bottom bands are almost dispersionless flat bands and share only one band touching point at the $\Gamma$ point. Further, the Fermi level is pinned right above the bottom flat band and the system becomes half-metallic semi-metal, when calculated without SOC. Interestingly, two conical bands and the nearly flat hole band near the zone center show typical Kane model type band alignment^45,46^. At *U* = 0 eV, the system shows typical three bands above Fermi level. At critical *U*, Kane fermion phase with triple degeneracy at $\Gamma$ point arises. By increasing *U* more, the system shows band inversion thereby to form four bands and the Fermi level crosses the meeting point of the upper cone and the flat band.

To provide the microscopic picture of the transition, we analyzed a symmetry of three involving bands. In Fig. 3(b,d), the *U* = 0 and 3.5 eV band structures are shown along $\Gamma -K$ direction in the Brillouin zone (BZ). At *U* = 0 eV the highest occupied flat band and second highest occupied conical band (Fig. 3(b)) have doubly degenerate eigenstates $|{e}_{1},\Gamma \rangle$, $|{e}_{2},\Gamma \rangle$ at $\Gamma$ point below Fermi level while a singly degenerate unoccupied eigenstate $|{a}_{1},\Gamma \rangle$ exist at $\Gamma$ point just above Fermi level. The *p*–*d* hybridization between Fe-d_*xz*_,d_*yz*_ and S-p_*z*_ is significant near Fermi level in majority spin channel, at *U* = 0 eV, as can be seen from the partial density of states plot for Fe *d* and S *p* orbitals. The molecular orbitals corresponding to singly degenerate and doubly degenerate eigenstates are plotted in figure Fig. 3(c). The $|{a}_{1},\Gamma \rangle$ has a node at Fe site with zero weight from d_*xz*_ + d_*yz*_ orbitals. On the other hand, molecular orbitals for doubly degenerate eigenstates $|{e}_{1},\Gamma \rangle$ has finite weight from Fe d_*xz*_ + d_*yz*_ orbitals. With the increase in Hubbard *U* to 3.5 eV at Fe site, the *p*–*d* hybridization gets lowered due to the localization of Fe *d* states below Fermi level. That makes zero weight for Fe-d_*xz*_,d_*yz*_ system above Fermi though S-p_*z*_ weight remain same. It makes the band inversion at $\Gamma$ point by making doubly degenerate eigenstates $|{e}_{1},\Gamma \rangle$, $|{e}_{2},\Gamma \rangle$ partially filled higher in energy and singly degenerate eigenstate $|{a}_{1},\Gamma \rangle$ fully occupied lower in energy. Thereby, the flat band makes a pair with above Dirac band as shown in Fig. 3(d).

This band inversion is responsible for making the system nontrivial with the inclusion of spin-orbit coupling. The Hubbard *U* = 0 eV band structure is trivial with respect to total Berry curvature up to the occupied level. It is also checked by edge state calculation. Especially, Chern number of the flat band is zero while the dispersive conduction band carries the non-zero Chern number *C* = 1 (see Fig. 3(e)). For *U* = 0 eV, through the band inversion, the conduction band transfers its Chern number to the flat band for *U* = 3 eV. As a result, the Chern number of the flat band as well as whole valence bands become *C* = 1. Our band structure and topological properties of MDT with smaller *U* (<2.4 eV) is consistent with the previous report^47^.

**Figure 4:**
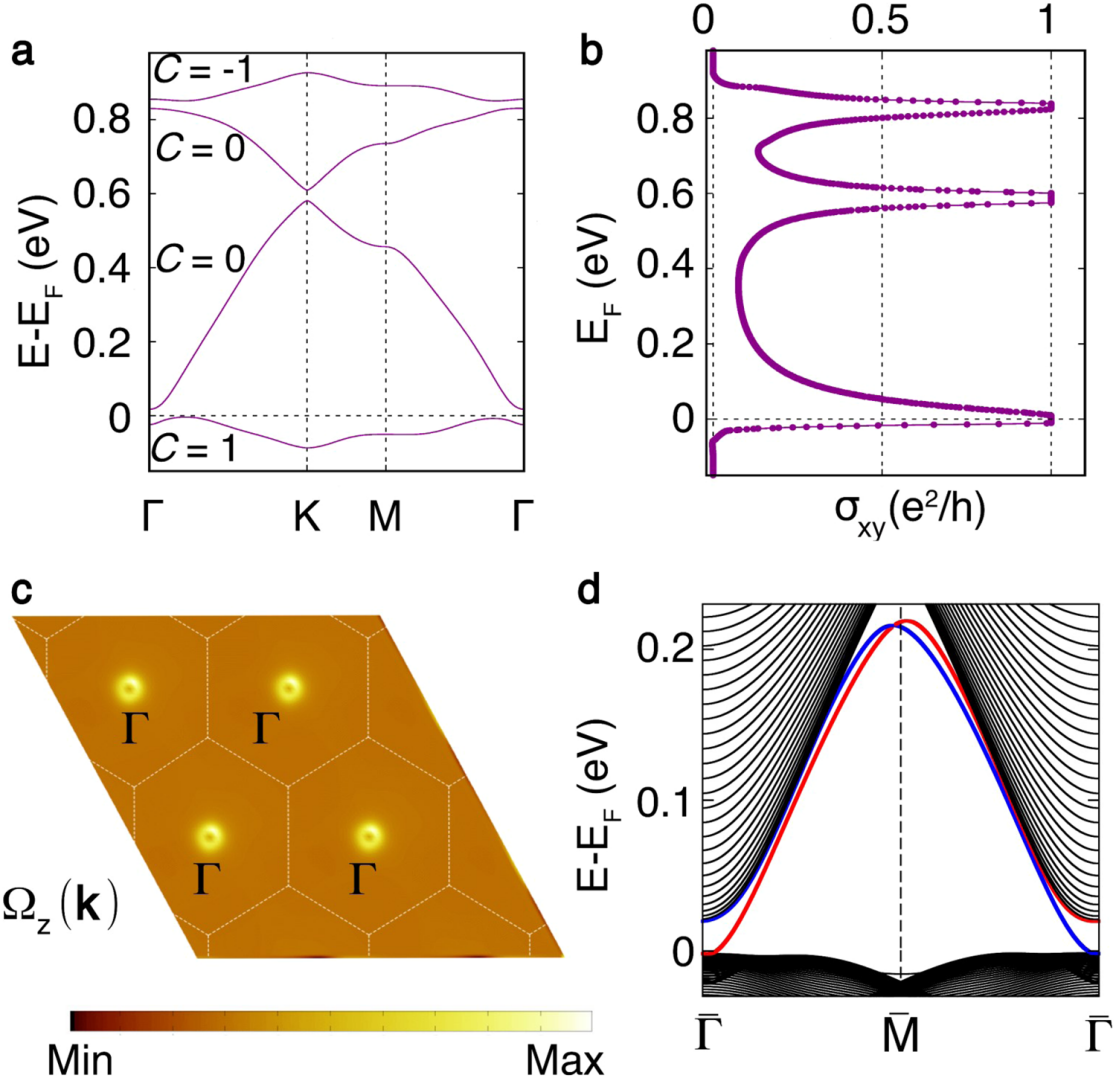
(**a**) HSE06 + SOC band structure (violet line) shows large band gap at Fermi level. The Chern number (*C*) of each band is also marked in the figure. (**b**) The anomalous Hall conductivity *σ*_*z*_ in unit of quantum conductance is shown with variation of Fermi level. (**c**) The Berry curvature in the BZ. (**d**) The left (red) and right (blue) edge bands of FDT.

To improve the estimation of a band gap, we further employed the HSE hybrid functional calculation as shown in Fig. 4. It opens a gap at the Fermi level as large as 22 meV for FDT complex and 23 meV for MDT complex (shown in Supplementary material). With this enhanced gap, we have used maximally localized Wannier functions (MLWFs) to calculate the anomalous Hall conductivity and Berry curvature by using the Wannier90 package^48^. The energy manifold chosen to accurately reproduce DFT bands (HSE06 + SOC) is set from −2.76 eV to 1.47 eV with respect to Fermi energy. The integration of the Berry curvature ${\Omega }_{z}(k)$ over the entire BZ gives anomalous Hall conductivity *σ*_*z*_. The variation of anomalous Hall conductivity *σ*_*z*_ as a function of energy is shown in Fig. 4(b). It clearly show that the dispersive conical bands above Fermi level are trivial though The unoccupied flat band is nontrivial. When we focus on the Berry curvature up to Fermi level, as shown in Fig. 4(c), the Berry curvature ${\Omega }_{z}(k)$ is concentrated only at $\Gamma$ point in the entire BZ. The quantized value of anomalous Hall conductivity shows Chern number *C* = 1 as Chern number is given by $C=\frac{1}{2\pi }\,{\int }^{}\,{d}^{2}{k\Omega }_{z}(k)$. The system, therefore, is a Chern insulator with highest occupied non-trivial nearly flat band with Chern number *C* = 1.

According to quantum transport theory, each edge current carries one *e*^2^/*h* conductivity. So, the direct confirmation of Chern insulating phase is to verify presence of chiral edge mode circulating at the boundary. The edge band structure was calculated using real space Hamiltonian in the basis of MLWFs and iterative Green’s function approach^49^. The edge band structure with left edge band (red) and right edge band (blue) connecting valence bulk band with conduction bulk bands is shown in Fig. 4(d). The number of chiral edge bands on one edge confirms the Chern number *C* = 1.

The presence of flat band in metal-organic framework based metal bis-dithiolene further pushes the organometallic system towards the direction of fractional quantum Hall effect. The redox control on these complexes are possible in practice as already reported in the previous literatures on Ni_3_C_12_S_12_^30^. So, with the proper control of electronic charge on highest occupied flat band exotic fraction Chern insulating phase can be realized in the future.

In summary we have shown that metal-organic framework based single layer metal bis-dithiolene complexes *M*_3_C_12_S_12_ (*M* = Mn and Fe) can be robust 2D ferromagnetic organic Chern insulator. From the DFT + *U* as well as hybrid-functional calculations, we confirmed that the Ising-type ferromagnetic state is the magnetic ground state of the single layer *M*_3_C_12_S_12_ with a gap as large as 22 meV. By controlling *U*, a topological quantum phase transition mediated by Kane fermion phase gives rise to a nearly flat band with a nonzero Chern number. To observe these phases in an experiment a wide band gap substrate would be necessary to avoid charge transfer between substrate and the compound near Fermi level. It would also be an interesting project to study the electronic and topological properties under the effect of strain. In addition, the presence of nontrivial flat band further pushes the boundary of metal-organic framework system towards realization of many-body phenomena like fractional Chern insulators by means of partial filling of the flat band with the help of any kind of hole doping.

## Methods

To determine the crystal structures and the electronic structures of the single-layer *M*_3_C_12_S_12_, we carried out first-principles density-functional-theory (DFT) calculations by employing both the PBE + *U* and the Heyd-Scuseria-Ernzerhof screened hybrid functional (HSE06)^50^ as implemented in the vasp^51,52^. All the calculations were performed with a plane-wave cutoff of 600 eV on the 6 × 6 × 2 Gamma centered Monkhorst-Pack *k*-point mesh in vasp. The OpenMX code^53^ based on the linear combination of pseudo-atomic-orbital basis formalism was used to do analysis for molecular orbitals. The energy cut-off for OpenMX is 400 Ryd. The vacuum layer was set to be 15 Å thick to decouple neighbouring layers along the *z*-direction. The full relaxation was carried out with keeping the three-fold rotational symmetry and inversion symmetry inherent in the space group P6/mmm with forces smaller than 0.01 eV/Å. The analysis of topological properties was carried out using Wannier90 package^48^ to fit tight-binding Hamiltonian in the basis of maximally-localized Wannier function (MLWFs) to DFT band structures.

## Supplementary information

Supplementary information

## Data Availability

All data in this work are available from the corresponding author on reasonable request.
