# Supplementary material for: Chern insulator with a nearly flat band in the metal-organic-framework-based Kagome lattice
Source: Sci Rep. 2019 Sep 24;9:13807. doi: 10.1038/s41598-019-50163-7 (PMC6760139; doi:10.1038/s41598-019-50163-7)
Supplement: Supplementary file 1 — Supplementary information [file 41598_2019_50163_MOESM1_ESM.pdf]

Supplementary Information for
"Chern insulator with a nearly flat band in the metal-organic-framework-based Kagome lattice"

Santu Baidya,[1,2] Seungjin Kang,[3] Choong H. Kim,[1,2,*] and Jaejun Yu[3,†]

[1]Center for Correlated Electron Systems, Institute for Basic Science, Seoul 08826, Korea
[2]Department of Physics and Astronomy, Seoul National University, Seoul 08826, Korea
[3]Center for Theoretical Physics, Department of Physics and Astronomy, Seoul National University, Seoul 08826, Korea

(Dated: July 8, 2019)

I. THE OPTIMIZED CRYSTAL STRUCTURE OF $M_3C_{12}S_{12}$ (M= MN AND FE)

The monolayer $M_3C_{12}S_{12}$ (M= Mn and Fe) is simulated by replacing Ni in monolayer $Ni_3C_{12}S_{12}$ with Mn and Fe. Both the structures were fully relaxed under PBE approximation using VASP package keeping the full symmetry. The Table S1 summarize the difference between two optimized crystal strcutures. The bond lengths d^{M-M} between nearest-neighbour M cations clearly shows C_3 symmetry of the system.

TABLE S1: Optimized geometry of $Fe_3C_{12}S_{12}$ and $Mn_3C_{12}S_{12}$

$Fe_3C_{12}S_{12}$, space group=P6/mmm (191), a=14.7988798 Å, β=120 deg						
Sites	wyckoff	x	y	z	d^{M-S} (Å)	d^{S-S} (Å)
Fe	g	0.0	0.5	0.0	7.400	3.090, 3.058
S	q	0.5447	0.6640	0.0		
C	q	0.2369	0.5705	0.0		
$Mn_3C_{12}S_{12}$, space group=P6/mmm (191), a=14.97807803 Å, β=120 deg						
Sites	wyckoff	x	y	z	d^{M-S} (Å)	d^{S-S} (Å)
Fe	g	0.0	0.5	0.0	7.489	3.165, 3.201
S	q	0.5439	0.6673	0.0		
C	q	0.2381	0.5716	0.0		

The bond length d^{S-S} between coordinated S ions around M cation show rectangular-plaquette unlike square-plaquette in isostructural $Ni_3C_{12}S_{12}$.

II. MONTE CARLO SIMULATION OF THE VARIATION OF MAGNETIZATION AND SPECIFIC HEAT WITH TEMPERATURE

Here, we address the computation of magnetic T_c using Mean field and Monte Carlo method. To calculate magnetic T_c for the classical spin of Fe $S = 1$ and Mn $S = 3/2$ we used the mean field exapression T_c=$\frac{zJS^2}{3k_B}$ due to the classical nature of the spins. The Collinear ferromagnetic and anti-ferromagnetic spin orientations are considered up to the nearest neighbours to calculate exchange interactions J which comes out to be $J_{Fe-Fe} \sim 11.3$ meV and $J_{Mn-Mn} \sim 4.2$ meV. The resulting magnetic T_c for Mn $T_c^{Mn} \sim 291$ K and for Fe $T_c^{Fe} \sim 352$ K. The calculation of the magnetic T_c is repeated with Monte Carlo method using ALPS[1] by computing variation of the magnetization ($|M|$) and specific heat (C_V) with temperature. From DFT calculation we find that the large SOC of S ions hybridized with Fe and Mn localized 3d spins pin the moments of Fe and Mn to lie along out-of-plane direction effectively behaving as ising spins. So, we have carried out Monte Carlo simulation of Heisenberg-Ising type Hamiltonian of classical spins of the honeycomb lattice of Fe and Mn ions. As in monte carlo simulation result may vary with increasing lenght of lattice we calculated for increasing lenght of lattice and found converged results. The specific heat in the Figure S1(a) and (c) clearly shows the paramagnetic to ferromagnetic transition for both Fe and Mn sites. The magnetic T_c at which specific heat shows the sharp peak for Mn $T_c^{Mn} \sim 234$ K and for Fe $T_c^{Fe} \sim 281$ are smaller than the mean-field estimate.

III. EDGE STATE PROJECTION FOR $FE_3C_{12}S_{12}$

To confirm the existence of Chern C number of edge state on each edge of the slab of $Fe_3C_{12}S_{12}$ we projected edge Green's function on left and right edge separately using Wanniertool[2] as shown in Fig S2. The figure shows exactly one edge state on both left and right of the slab confirming chern number $C = 1$.

IV. ENERGY COMPARISON OF COLLINEAR AND NONCOLLINEAR SPIN CONFIGURATION OF $MN_3C_{12}S_{12}$ AND $FE_3C_{12}S_{12}$

The Table S2 shows the energy comparison of few spin configurations under PBE+U+SOC ($U \sim 3$ eV) of the $Fe_3C_{12}S_{12}$ and $Mn_3C_{12}S_{12}$. In both compounds the out-of-plane ising type ferromagnetiic ordering appears to be lower in energy.

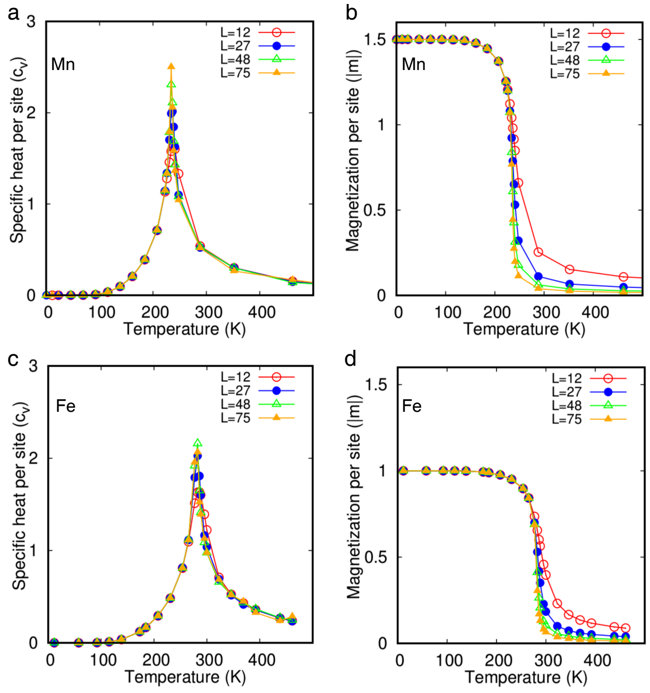

FIG. S1: (Color online) Monte Carlo simulation results for magnetic T_c of $M_3C_{12}S_{12}$ for various choices of number of lattices (a) variation of the specific heat with temprature for Mn ($S = \frac{3}{2}$), (b) magnetization with temprature for Mn ($S = \frac{3}{2}$), (c) variation of the specific heat with temprature for Fe ($S = 1$), and (d) magnetization with temprature for Mn ($S = 1$)

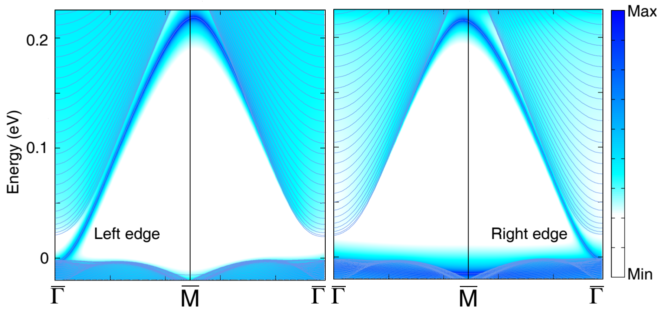

FIG. S2: (Color online) The edge state projection for $Fe_3C_{12}S_{12}$ at left and right edge of the slab under HSE06+SOC.)

V. ELECTRONIC STRUCTURE COMPARISON OF $MN_3C_{12}S_{12}$ AND $FE_3C_{12}S_{12}$

In the main article we have mentioned that the electronic structure of the two compounds MDT and FDT are similar. To clarify the statement we have compared the PBE+U bands structure of MDT and FDT in the Figure S3. Majority spin channel of both the compounds, MDT ($d^5L^{-2,\downarrow}$) and FDT ($d^6L^{-2,\downarrow}$), are completely filled, while electron filling can only happen in the minority spin channel. To replace Mn by Fe requires additional number of electrons to occupy the valence band. With the simplest possible explanation of rigid band shift we can see that in case of FDT additional electrons occupy the valence band in the minority spin channel giving rise to a rigid band shift in the minority bands, while there is almost no change in the majority bands. As the four bands near Fermi level correspond to the majority spin channel, there is almost no change in the electronic and topological properties of the two compounds. But, we do agree that due to the different electron filling the overall electronic properties of both the compounds should be different.

TABLE S2: PBE+U+SOC ($U \sim 3$ eV) energy comparison (in meV) of spin-configurations of $Fe_3C_{12}S_{12}$ (FDT) and $Mn_3C_{12}S_{12}$ (MDT)

System	FM(001)	FM(110)	AIAO (in-plane)
FDT	0.0	0.97	94.87
MDT	0.0	1.87	70.03

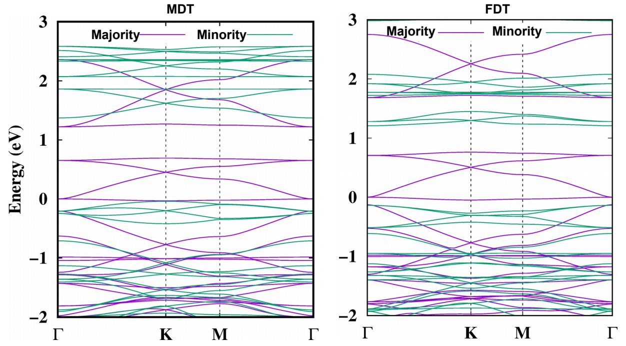

FIG. S3: (Color online) The PBE+U ($U \sim 3$ eV) eletronic bands of the and $Mn_3C_{12}S_{12}$ (MDT) (left) and $Fe_3C_{12}S_{12}$ (FDT) (right).

VI. PHONON DISPERSION OF $FE_3C_{12}S_{12}$ FOR DYNAMIC STABILITY

As we have mainly discussed about the $Fe_3C_{12}S_{12}$ (FDT) compound in the main text so in the Figure S4 the phonon dispersion is plotted. It shows no imaginary phonon modes indicating the dynamic stability of the compound.

* chkim82@snu.ac.kr
† jyu@snu.ac.kr
1 B. Bauer *et. al.*, J. Stat. Mech. P05001 (2011).
2 QuanSheng Wu, ShengNan Zhang, Hai-Feng Song, Matthias Troyer, Alexey A. Soluyanov, Computer Phys. Comm. 224, 405 (2018).

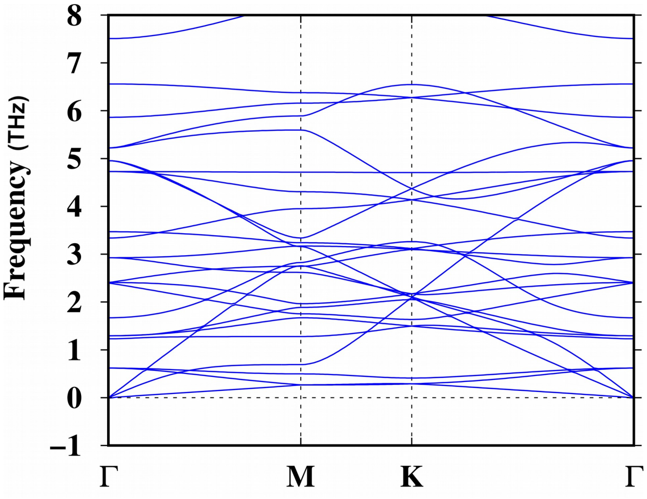

FIG. S4: (Color online) The phonon dispersion for $Fe_3C_{12}S_{12}$ showing no imaginary phonon modes.
